# Impact of positive surgical margin location and perineural invasion on biochemical recurrence in patients undergoing radical prostatectomy

**DOI:** 10.1186/s12957-020-01977-7

**Published:** 2020-08-13

**Authors:** Zhenpeng Lian, Hongtuan Zhang, Zhaowei He, Shenfei Ma, Xiaoming Wang, Ranlu Liu

**Affiliations:** Department of Urology, Tianjin Institute of Urology, The Second Hospital of Tianjin Medical University, Tianjin, 300211 China

**Keywords:** Positive surgical margins, Location, Perineural invasion, Biochemical recurrence, Prostate cancer

## Abstract

**Objective:**

To estimate the prognostic value of positive surgical margins (PSM) location and perineural invasion (PNI) for biochemical recurrence (BCR) in patients undergoing radical prostatectomy (RP).

**Methods:**

All men with prostate cancer (PCa) who received RP in the second hospital of Tianjin Medical University from 2014 to 2018 were retrospectively identified. All patients met the following criteria: no neoadjuvant or adjuvant treatment, absence of lymph node invasion, or distant metastasis confirmed by surgery or imaging. Comparisons were made between cases with only apex positive (AM), isolated nonapical positive (OM), multiple positive (MM), and negative surgical margins (NSM). Patients were also subdivided according to the Gleason score and pathological tumor stage for analysis.

**Results:**

A total of 416 patients available for analysis, of which 132 (31.7%) were PSM, 43 were AM, 37 were OM, and 52 were MM at a median follow-up of 27 months. The PNI was in 30.5% of patients. BCR occurred in 22.6% of patients during follow-up. Both AM and MM were noticed to be independent predictors of BCR with a hazard ratio of 4.192 (95% CI 2.185–8.042; *p* < 0.001) and 2.758 (95% CI 1.559–4.880; *p* < 0.001), respectively, when compared to NSM. Though the correlation was significant in univariate analysis, PNI was not an independent risk factor for BCR (*p* = 0.369). Subgroup analyses suggested that MM was not particularly predictive for BCR in the Gleason score < 8. The hole Cox regression model for the C-index was 0.843

**Conclusions:**

PSM location was a significant independent predictor of BCR in PCa, especially in patients with AM or MM, while PNI is a non-independent risk factor. Compared with other locations, AM has a higher BCR risk.

## Background

Positive surgical margin (PSM) after radical prostatectomy (RP) for prostate cancer (PCa) has been consistently considered an effective predictor of postoperative biochemical recurrence (BCR) [1–4]. Owing to patient selection, the experience of surgeons, surgical technique, and pathological specimen analyses, the incidence of PSM varies obviously between different researches and ranges from 6.5 to 38.4% [5–8]. The risk of PSM was reported to be related to high serum prostate-specific antigen (PSA), low prostate volume, high Gleason score (GS), and interfascial nerve [9]. The number, length, and GS of PSM have been followed in previous discussions. However, the impact of PSM location on BCR was reported several and remains controversial [10–12].

Perineural invasion (PNI) was defined as the trajectory of tumor cells along or around nerve fibers, which has been a recognized mechanism of tumor spread [9]. The existence of PNI is related to the adverse outcome for several malignancies, while the clinical significance of PNI was still controversial. Previous studies have shown that PNI is a predictor of adverse pathological and clinical features, as well as a strong predictor of BCR in PCa [13]. However, as a predictor of BCR, the independent value of PNI has not yet been established.

The aim of our study is to discuss the correlation of PSM location and PNI on the prediction of BCR for PCa (pathological stage T2–T3 patients), as well as those impacts on different subgroups.

## Patients and methods

### Patient selection

From 2014 to 2018, 416 patients of PCa who underwent RP in the second hospital of Tianjin Medical University were included. All patients met the following criteria: no neoadjuvant or adjuvant treatment, absence of lymph node invasion, or distant metastasis confirmed by surgery or imaging. The surgical techniques for RP differed among patients: open RP or laparoscopic RP. Both interventions were conducted by two experienced surgical teams. BCR was defined as a serum PSA level ≥ 0.2 ng/mL twice 3 months after RP. Early salvage therapy was conducted in patients with BCR. This study was approved by the local ethics committee.

### Pathology analysis

The Stanford protocol and Gleason system were used to processing all RP specimens. All pathologic specimens were step-sectioned for complete evaluation tumor grade, volume, and margins. The definition of PSM was the presence of definite tumor cells on the edge of the RP specimen [8]. In this study, we divided PSM locations into the apex, peripheral (included anterior, posterior, lateral), and base firstly. Negative surgical margins (NSM), a solitary positive apical margin (AM), a solitary nonapical positive margin (OM), a solitary positive margin (AOM), and multiple positive surgical margins (MM) were subdivided by margin status. Whenever two or more of these locations were positive, the PSM was deemed as MM.

### Statistical analysis

Frequencies and proportions were used to describe statistics of categorical variables. Interquartile ranges (IQR) and medians were presented for continuous variables. The Kruskal-Wallis *H* test and Chi-square test or Fish exact test were performed in continuous variables and categorical variables for statistical analysis. Kaplan-Meier survival analysis was conducted to evaluate the probability of BCR-free survival. A comparison of survival distributions was performed with the log-rank test or Breslow test. A backward stepwise multivariable cox regression analysis (entry-level at *p* ≤ 0.1) was modeled to evaluate BCR. Spearman’s rank was used for the correlations analysis, using Harrell consistency Index (c-index) to evaluate the discriminant ability of PSM and PNI state models. All analyses were performed with IBM SPSSv.23.0 and Stata15. All *p* values were bilateral, with *p* values < 0.05 considered statistically significant.

## Result

Table 1 summarizes patients’ characteristics of the 416 cases. Among the patients included in the study, 132 (31.7%) patients exhibit PSM. Of these, 43 (10.3%) were reported to have AM, 37 (8.9%) were OM, and 52 (12.5%) were MM. The median age and BMI were 68 and 24.9 kg/m2, respectively. The median prostate volume of patients was 24.6 ml and the median preoperative PSA was 15.8 ng/ml. In total, 152 (37%) patients were conducted by open RP, and the remainder used laparoscopic RP. The rate of BCR was 36.2% in PNI patients versus 16.6% in without PNI patients. When compared to a PSM with NSM cases, PSM cases revealed a higher preoperative PSA, GS, pathological tumor stage (pT), and PNI. The median follow-up time was 27 months (IQR 20–47). During follow-up, BCR occurred in 22.6% cases.

**Table 1:** Clinical and histological characteristics of patients according to the location of positive surgical margin (PSM)

Characteristics	Total	Surgical margin				***p*** value
		NSM	AM	OM	MM	
Patients, no. (%)	416 (100)	284 (100)	43 (100)	37 (100)	52 (100)	-
Age, years, median (IQR)	68 (62–72)	67 (62–72)	68 (63–71)	68 (64–71)	68 (61–75)	0.922
BMI, kg/m2, median (IQR)	24.9 (23.5–27.0)	25.0 (23.7–27.1)	24.7 (23.8–27.1)	25.1 (22.9–27.0)	24.0 (22.9–27.0)	0.078
Volume, ml, median (IQR)	24.6 (18.7–34.6)	24.6 (18.7–36.1)	19.5 (16.6–28.1)	25.7 (18.1–36.3)	26.4 (16.4–35.9)	0.167
PSA, ng/ml, median (IQR)	15.8 (8.8–29.8)	13.1 (8.3–25.7)	15.8 (7.3–25.6)	26.0 (13.8–57.0)	22.6 (14.0–55.9)	< 0.001
Surgical approach, no. (%)						
ORP	152 (37)	125 (44)	8 (19)	12 (32)	7 (13)	< 0.001
LRP	264 (63)	159 (56)	35 (81)	25 (68)	45 (87)	
Gleason score, no. (%)						
< 8	246 (59)	193 (68)	25 (58)	14 (38)	14 (27)	< 0.001
≥ 8	170 (41)	91 (32)	18 (42)	23 (62)	38 (73)	
pTNM, no. (%)						
T2	270 (65)	187 (66)	34 (79)	15 (41)	34 (65)	0.004
T3	146 (35)	97 (34)	9 (21)	22 (59)	18 (35)	
Perineural invasion, no. (%)						
No	289 (69)	213 (75)	30 (70)	22 (59)	24 (46)	< 0.001
Yes	127 (31)	71 (25)	13 (30)	15 (41)	28 (54)	
BCR						
No	322 (77)	238 (84)	30 (70)	24 (65)	30 (58)	< 0.001
Yes	94 (23)	46 (16)	13 (30)	13 (35)	22 (42)	
Follow-up time, median (IQR)	27 (20–47)	32 (23–44)	23 (19–29)	25 (17–31)	19 (16–26)	< 0.001

*NSM* negative surgical margins, *AM* a solitary positive apical margin, *OM* a solitary nonapical positive margin, *MM* multiple positive surgical margins, *PSA* prostate-specific antigen, *BCR* biochemical recurrence

Regarding all PSM locations, the most common was the apex, exhibited in 82/191 cases. For the peripheral and base, 53/191 and 56/191 cases exhibited those locations respectively (data not shown). In cases with PSM, 36.3% develop BCR compared with just 16.2% in NSM. AM, OM, and MM cases had higher rates of BCR (30.2%, 35.1%, and 42.3%, respectively) than those with NSM. Among them, MM experienced BCR earlier than the AM and OM.

**Fig. 1:**
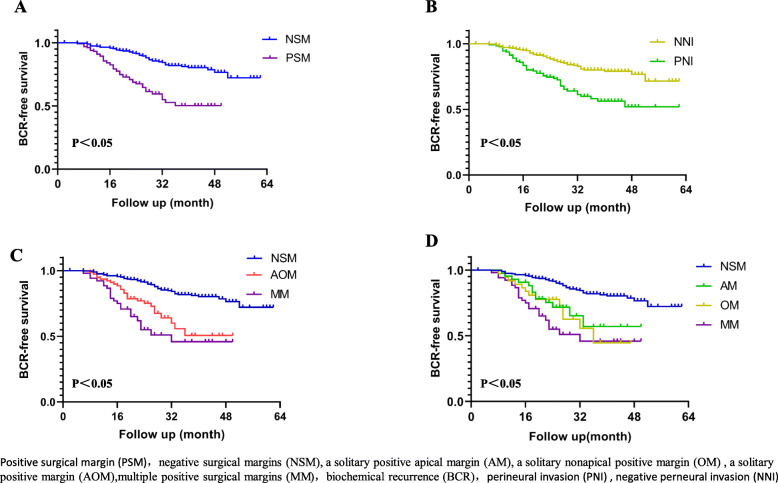
Kaplan-Meier curves showing biochemical recurrence (BR)-free survival following. **a** PSM and NSM. **b** PNI and NNI. **c** NSM, AOM, and MM. **d** NSM, AM, OM, and MM

BCR-free survival at 3 years was 81.9% for patients with NSM versus 50.3% for those with PSM (*p* < 0.001), 51.5%, 57.1%, 44.6%, and 45.9% for AOM, AM, OM, and MM, respectively (*p* < 0.001). As for the patients with PNI, BCR-free survival at 3 years was 58.2% compared to 80% without PNI (*p* < 0.05, Fig. 1). We discovered that patients with PSM and PNI were more likely to develop BCR.

The results of univariable and multivariable cox regression analyses for predicting PSM are shown in Table 2. In the univariable analysis, PSA level, GS, pT, surgical margin, and the PNI were all significant risk factors for a BCR (*p* < 0.001). AM, OM, and MM were statistically significant in univariable analysis, while in a multivariable analysis, only AM and MM remained independently significant predictors for BCR. The BCR risk between AM and NSM increased by 4.192 times (95% CI 2.185–8.042; *p* < 0.001), and that between MM and NSM increased by 2.758 times (95% CI 1.559–4.880; *p* < 0.001). There was also a connection between pT and BCR: pT3 were associated with a higher risk of recurrence compared to pT2 (HR = 2.526, 95% CI 1.614–3.955; *p* < 0.001), as well as GS < 8 with a lower risk of recurrence compared to GS ≥ 8 (HR = 4.629, 95% CI 2.718–7.883; *p* < 0.001). The preoperative PSA level was also significantly associated with BCR (HR = 1.012, 95% CI 1.009–1.016; *p* < 0.001). It was worth to be noted that BMI or PNI was not an independent predictor for BCR, though PNI was statistically significant in univariable analysis.

**Table 2:** Univariate and multivariate model and hazard ratio calculations for variables associated with biochemical recurrence

Variables	Univariable cox regression		Multivariable cox regression (backward stepwise)	
	HR (95% CI)	***p*** value	HR (95% CI)	***p*** value
Age (years)	0.993 (0965–1.023)	0.646		
BMI (kg/m^2^)	0.939 (0.875–1.008)	0.081	0.975 (0.906–1.050)	0.508
Volume(ml)	1.005 (0.995–1.015)	0.349		
TPSA (ng/ml)	1.016 (1.013–1.018)	**< 0.001**	1.012 (1.009–1.016)	**< 0.001**
Surgical approach				
ORP	1.000 (Ref.)	0.979		
LRP	1.006 (0.665–1.521)			
Gleason score				
< 8	1.000 (Ref.)	**< 0.001**	1.000 (Ref.)	**< 0.001**
≥ 8	7.796 (4.702–12.925)		4.629 (2.718–7.883)	
pT stage				
T2	1.000 (Ref.)	**< 0.001**	1.000 (Ref.)	**< 0.001**
T3	3.239 (2.136–4.911)		2.526 (1.614–3.955)	
Surgical margin		**< 0.001**		**< 0.001**
NSM	1.000 (Ref.)		1.000 (Ref.)	
AM	2.687 (1.443–5.005)	0.002	4.192 (2.185–8.042)	< 0.001
OM	3.147 (1.689–5.862)	<0.001	1.501 (0.794–2.838)	0.211
MM	4.530 (2.702–7.595)	<0.001	2.758 (1.559–4.880)	< 0.001
Perineural invasion				
No	1.000 (Ref.)	**< 0.001**		0.369
Yes	2.503 (1.669–3.753)		1.219 (0.791–1.877)	

In spearman’s rank-order correlation analysis for PNI, there was a significant correlation of PNI with GS and pT (r1 = 0.277, r2 = 0.267). Subgroup analysis for BCR, PSM, and AM remained independent predictors in both GS and pT stage group. Neither OM nor PNI was predictive for BCR after patients adjusted for the variable in a multivariable cox regression analysis. OM was even not a predictor for BCR in the pT2 group (Tables 3 and 4).

**Table 3:** Cox regression analysis evaluating the value of surgical margins and perineural invasion as a predictor of time to biochemical recurrence in a subgroup analysis regarding Gleason Score

Variable	Gleason Score < 8 (***n*** = 246)				Gleason Score ≥ 8 (***n*** = 170)			
	Univariable		Multivariable^**1**^		Univariable		Multivariable^**1**^	
	HR (95% CI)	***p*** value	HR (95% CI)	***p*** value	HR (95% CI)	***p*** value	HR (95% CI)	***p*** value
Surgical margins		**0.041**		**0.033**		**0.002**		**0.001**
NSM	1.0 (Ref.)		1.0 (Ref.)		1.0 (Ref.)		1.0 (Ref.)	
AM	4.4 (1.5–12.7)	0.006	5.1 (1.7–14.7)	0.003	2.2 (1.0–4.9)	0.049	3.8 (1.6–8.7)	0.002
OM	-	0.984			2.2 (1.2–4.4)	0.012	1.6 (0.8–3.1)	0.164
MM	3.0 (0.7–13.7)	0.15	2.0 (0.4–9.2)	0.394	2.9 (1.6–5.1)	<0.001	3.0 (1.6–5.6)	0.001
Perineural invasion								
No	1.0 (Ref.)		1.0 (Ref.)		1.0 (Ref.)		1.0 (Ref.)	
Yes	1.4 (0.5–3.8)	0.561			1.8 (1.1–2.8)	**0.016**	1.2 (0.7–1.9)	0.493

^1^Adjusted for BMI, TPSA, and pathological tumor stage

**Table 4:** Cox regression analysis evaluating the value of surgical margins and perineural invasion as a predictor of time to biochemical recurrence in a subgroup analysis regarding pathological tumor stage

Variable	pT2 (***n*** = 270)				pT3 (***n*** = 146)			
	Univariable		Multivariable^**1**^		Univariable		Multivariable^**1**^	
	HR (95% CI)	***p*** value	HR (95% CI)	***p*** value	HR (95% CI)	***p*** value	HR (95% CI)	***p*** value
Surgical margins		**< 0.001**		**0.002**		**< 0.001**		**0.011**
NSM	1.0 (Ref.)		1.0 (Ref.)		1.0 (Ref.)		1.0 (Ref.)	
AM	4.8 (2.1–11.2)	< 0.001	4.9 (2.1–11.8)	< 0.001	2.3 (0.8–6.5)	0.122	3.7 (1.2–11.1)	0.019
OM	2.1 (0.5–9.1)	0.343	2.6 (0.6–11.5)	0.216	2.6 (1.3–5.3)	0.007	1.3 (0.6–2.6)	0.51
MM	6.5 (3.0–14.5)	< 0.001	3.1 (1.3–7.5)	0.014	4.5 (2.2–9.2)	< 0.001	2.8 (1.4–5.9)	0.005
Perineural invasion								
No	1.0 (Ref.)				1.0 (Ref.)		1.0 (Ref.)	
Yes	2.4 (1.2–4.6)	**0.01**	0.9 (0.4–2.0)	0.808	1.7 (1.0–2.9)	**0.044**	1.5 (0.9–2.6)	0.157

^1^Adjusted for BMI, TPSA, and Gleason Score

The prediction accuracy of the COX regression model for NSM, AM, OM, and MM stratification was 0.843. When surgical margin location status or PNI was removed from the hole model, the C-index was 0.827 or 0.841, respectively.

## Discussion

In the present large, single-center study of RP cases, we have shown that patients with AM and MM showing as an independent predictor of BCR when compared to patients with NSM on multivariate analysis. Patients with AM were more important for the prediction of BCR relative to patients with MM and OM. Interestingly, patients with PNI and OM exhibited significantly worse BCR prognosis in univariable analysis; however, this significance was lost on multivariate analysis. The same conclusion was conducted by subgroup analysis. BR-free survival at 3 years was 81.9% for patients with NSM versus 50.3% for those with PSM. The BR-free survival rates seemed to be lower than in previous studies. Preisser et al. who identified 8770 patients suggested that the 72-month BCR-free survival rates of PSM and NSM group after RP were 77.7% and 89.0%, respectively [14]. A potential explanation may be the patient’s selection. In our study, patients with high stage and GS occupied a considerable number. All patients were not accepted adjuvant treatment before BCR. As for the patients with PNI, the BR-free survival rates were 80% versus 58.2%. Similar conclusions could be found in previous studies [15].

Though PSM revealed a significant hazard ratio by adjusted cox regression analysis, the location result from RP is not equivalent to BCR. Our study suggested that BCR was independently associated with AM and MM but not with OM, in addition to BMI, PSA level, GS, pT stage, and PNI. The AM appeared to have greater effects across all margin locations and a significant contributor to BCR across all statistical models, particularly when compared to OM: 4.192 versus 1.501. However, the significance of PSM location on BCR was still controversial in the current literature, especially in AM. In a large series of 4001 patients, Dev et al. suggested that the apex is an important factor affecting BCR, and it seems to be the strongest among all the risk factors [16]. Choo et al. and Porpiglia et al. also found that positive apical is a significant risk factor of BCR compared with nonapical margins [17, 18]. Nevertheless, Gautier et al. showed that independently from the pT stage, GS, and lymph nodes invasion, focal apical PSM had no significant effect on BCR, while extensive apical PSM significantly increased the risk of BCR [19].

Further research on PSM patients revealed that apex was the most common PSM site for RP. This may be owing to its location that closes to the dorsal venous plexus and neurovascular bundles plexus with a little capsule. Furthermore, the surgeons also attempt to conserve maximum urethral length, in order to protect the sexual and urine function better [10]. The possible reason for AM being more likely to BCR may also be caused by its location with abundant blood supplement. In addition to the special location, some patients with AM suggested that the tumor might invade part of the urethra with the epithelium of transitional cells, which is metastasis and recurrence. Our study also discovered that AM patients’ prostate volume was less than others. Recent research found men with smaller prostates were at greater risk of progression after RP. For a given age, the small prostate may be associated with lower androgen levels and the total concentration of growth factors in the prostate. Owing to PSA-driven biopsies, men with larger prostates can detect their tumors earlier [20]. Those may explain why we should focus on patients with AM.

We also demonstrated the greater impact of PSM on BCR after subgroup analysis, especially in AM. The correlation between OM and PNI was still found in univariable analysis. Interestingly, after adjusting for BMI, PSA level, and GS, we could not find any significant association between MM and BCR in GS < 8 group. In addition to the lack of statistical efficiency, one possible explanation may be that MM was only a significant predictor for BCR in high-risk PCa.

Preoperative multi-parametric MRI may play an important role in the staging and surgical planning of PCa, and it may also have an impact on PSM. For the patients with TNM stage < T3, the RP can be a curative treatment. However, for patients with extracapsular extension, seminal vesicle invasion, and distant metastasis, the preoperative MRI evaluation of tumor stage mainly determine the surgical planning and affect the prognosis of patients [21]. A study involved 353 patients showed that the initial surgical plan was changed in 26% of patients after the surgery reviewed MRI images. Of these patients, the majority was intermediate or high-risk groups and 90% of patients chose a more conservative operation [22]. Jaderling et al. investigated 557 patients demonstrated that MRI examination would increase the rate of non-nerve sparing operation and reduce PSM, while Rud et al.’s research observed a possible benefit of MRI in patients only with T1 [23, 24].

Different surgical planning and technique may also have an impact on the surgical margin during RP. According to the mode of operation, RP can be divided into bilateral nerve sparing, unilateral, or non-nerve sparing. Previous studies have found that compared with unilateral or non-nerve sparing, bilateral nerve sparing patients had better sexual and urinary function especially in terms of men with good baseline sexual function [25]. Preston et al. evaluated 6120 patients who underwent open, laparoscopic, or robotic RP with the stage from T2 to T3 and demonstrated that there was a significant difference between robotic and open RP, while the bilateral nerve sparing was only associated with increased risk of PSM in the stage of T2. They assumed that nerve sparing should be performed in patients with a good sexual function and patients who could accept the risk of PSM after the operation [26]. A similar study conducted by Atsushi et al. also showed that the PSM rates were 27.6% (open), 18.4% (laparoscopic), and 13.4% (robotic), respectively, and surgical approach (open vs robot) were independent risk factors for PSM [6]. In our study, all patients were evaluated for sexual function before the operation. Nerve sparing therapy would be conducted in patients with earlier stage and better physical condition. For patients with and without nerve preservation, the surgical dissection carried above and below Denonvillier’s fascia, respectively. Open and laparoscopic RP patients also found a significant difference in PSM (*p* < 0.001).

Our results showed that 30.5% of patients had PNI, while the incidence in other research ranged from 21.1 to 50.2% [13, 27–30]. The variation between these studies may be associated with different patient groups or pathological criteria. Though the patients with PNI had reported that short BCR-free survival time and advanced pathological outcomes were observed in some studies, the relationship between PNI and BCR after RP is still controversial [13, 15, 29]. Kang et al. found that PNI is an important predictor in PCa patients treated with RP; however, its prognostic value has not been detected in localized PCa [28]. Jung et al. reported that PNI was independently related to the aggressive pathological features of PCa, while it did not predict BCR [31]. Loeb et al. also claimed that PNI was not an independent risk factor for BCR after RP, while it was still an independent factor of advanced pathology features in multivariate analysis [32]. The results above were in line with our findings. Although our study discovered a significant correlation between PNI and BCR in univariable analysis, after adjusting for BMI, PSA level, GS, and pT stage, the relationship was no longer statistically significant. A similar conclusion was observed in the subgroup analysis. Patients with PNI in GS < 8 even could not find a correlation with BCR in univariable analysis.

In our study, the higher risk of BCR also may occur if a PSM is present with a higher PSA level, pT3, and GS ≥ 8. Preisser et al. and Max Kates et al. illustrated that a higher GS at the PSM is independently associated with early BCR. In these cases, adjuvant therapy should be considered after RP [14, 33].

The study also has some limitations. First, there is no consistent record of PSM length in the available pathological reports and further study could not be analyzed. Secondly, we have a relatively short follow-up period. Further follow-up of these patients should be continued for disease progression or overall survival. Finally, large-scale multicenter studies were still needed to illustrate the exact prognostic value of PSM location and PNI in PCa patients.

## Conclusions

We recommend careful evaluation of patients with PSM location following RP, especially if they are AM or MM with higher PSA level, pT3, and GS ≥ 8. In these cases, adjuvant therapy should be considered after radical surgery.

## Data Availability

The datasets used and/or analyzed during the current study are available from the corresponding author on reasonable request.

## Abbreviations

PSM: Positive surgical margin
RP: Radical prostatectomy
PCa: Prostate cancer
BCR: Biochemical recurrence
GS: Gleason score
PSA: Prostate-specific antigen
PNI: Perineural invasion
AM: Solitary apical positive margin
OM: Solitary nonapical positive margin
MM: Multiple positive surgical margins
AOM: Solitary positive margin
IQR: Medians and interquartile
pT: Pathological tumor stage
